# Pulmonary artery rupture after bilateral pulmonary artery banding in a neonate with Loeys–Dietz syndrome and an interrupted aortic arch complex: report of a case

**DOI:** 10.1007/s00595-014-0910-8

**Published:** 2014-05-11

**Authors:** Hideto Ozawa, Hiroaki Kawata, Shigemitsu Iwai, Sanae Yamauchi, Tomomitsu Kanaya, Hidefumi Kishimoto

**Affiliations:** 1 Department of Cardiovascular Surgery, Osaka Medical Center, and Research Institute for Maternal and Child Health, Izumi, Osaka Japan; 2 Department of Cardiovascular Surgery, Osaka University Graduate School of Medicine, 2-2 Yamada-oka, Suita, Osaka 565-0871 Japan

**Keywords:** Loeys–Dietz syndrome, Pulmonary artery rupture, Bilateral pulmonary artery banding

## Abstract

Loeys–Dietz syndrome (LDS) is a recognized connective tissue disorder characterized by progressive aortic aneurysm and dissection. Patients are at high risk of aortic dissection or rupture at an early age, but to our knowledge, surgery on the great arteries has never been attempted in the neonatal period. We report a case of LDS with dilated pulmonary arteries and an interrupted aortic arch complex in a neonate. We performed bilateral pulmonary artery banding, but 12 days after the procedure, the infant died of rupture of the distal portion of the banding sites following massive dilatation.

## Introduction

Loeys–Dietz syndrome (LDS) has recently been recognized as a connective tissue disorder, caused by heterozygous mutations in the transforming growth factor-β genes, *TGFBR1* and *TGFBR2,* and characterized by aggressive aortic aneurysm and dissection [1]. Although affected patients are at high risk of aortic dissection or rupture at an early age, there are few reports of cardiovascular surgery being performed on neonates with LDS. We report a case of bilateral pulmonary artery (PA) banding for LDS with a double outlet right ventricle (DORV) and an interrupted aortic arch (IAA) in a neonate.

## Case report

**Fig. 1:**
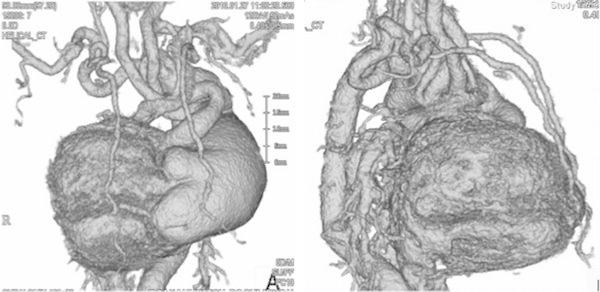
Enhanced computed tomography showed main pulmonary artery aneurysm and tortuosity of the cervical arteries

**Fig. 2:**
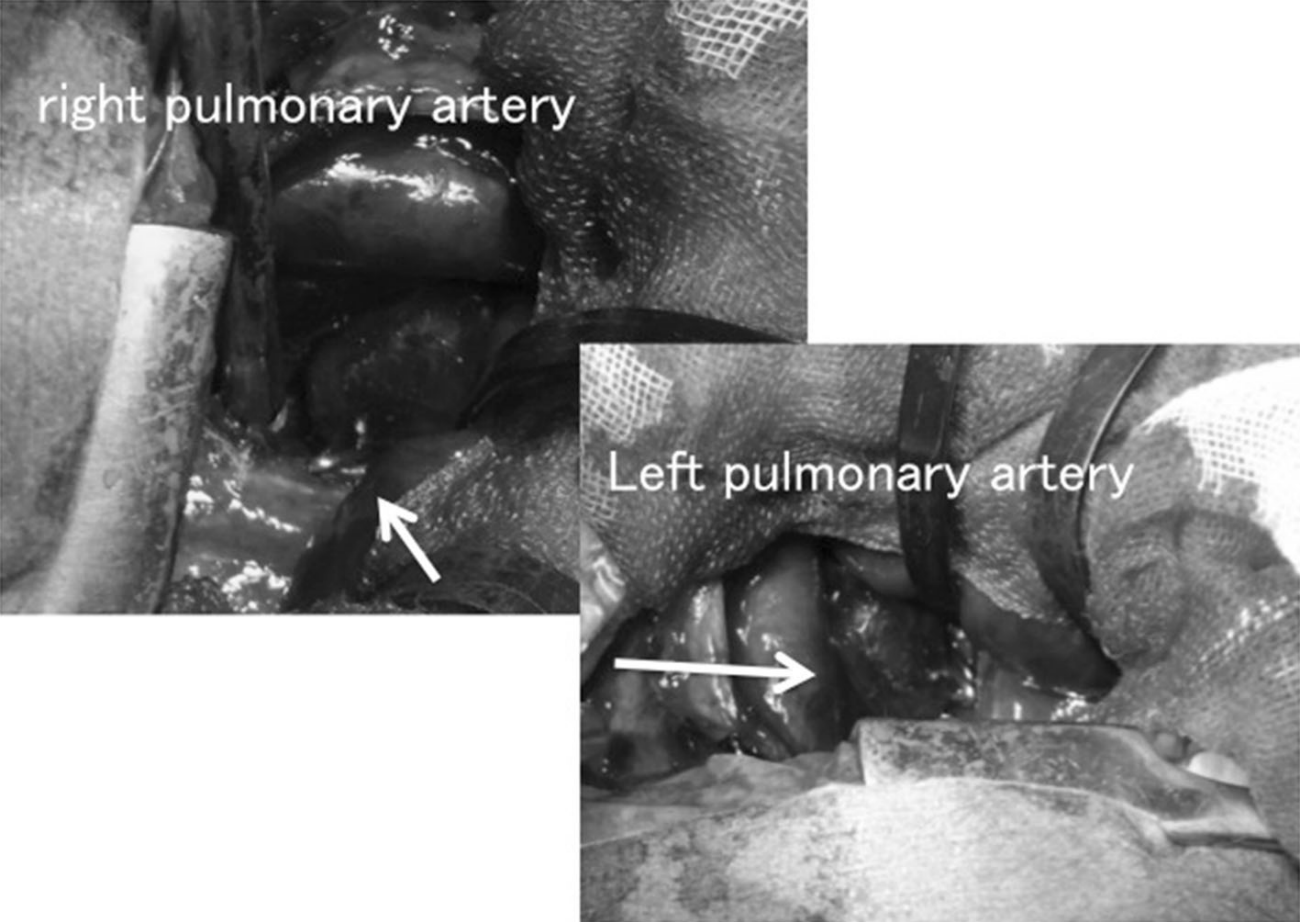
We used a hemoclip (*arrows*) as a partial clamp, instead of taping, to control the bleeding. Color Doppler showed a blood velocity of 3.0 m/s in the right PA banding site, suggesting that the banding was effective

**Fig. 3:**
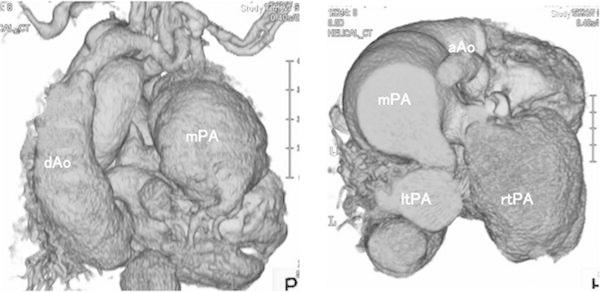
Enhanced computed tomography showed significant dilatation of the bilateral pulmonary arteries and descending aorta. *mPA* main pulmonary artery, *rtPA* right pulmonary artery, *ltPA* left pulmonary artery, *dAo* descending Aorta

The patient, a male infant, was the first-born child of healthy non-consanguineous Japanese parents. In the 36th week of gestation, the fetus was found to have a DORV, IAA, and a huge aneurysm in the main PA. The baby was delivered by caesarian section in the 39th week of gestation, with a birth weight of 3,542 g. Soon after delivery, dyspnea developed and the infant was intubated. He also had a cleft palate, bifid uvula, odd facial appearance, joint laxity, and translucent skin. Echocardiography indicated DORV with a subpulmonic ventricular septal defect (VSD), IAA, and patent ductus arteriosus (PDA). Enhanced computed tomography showed a main PA aneurysm and tortuosity of the cervical arteries (Fig. 1). This manifestation was highly suggestive of LDS. We suspected that the patient’s arterial tissue would be too fragile for repair of the interrupted arch and VSD; therefore, we decided to perform bilateral PA banding, the most minimally invasive surgery in this circumstance, when the infant was 2 weeks of age. However, at 9 days of age, the patient suffered a right pulmonary hemorrhage caused by high pulmonary blood flow, necessitating emergency bilateral PA banding. Since both PAs arise from behind the huge main PA, we accessed each of them via bilateral thoracotomy. We performed the right PA banding first to reduce the right PA hemorrhage. The right PA was 9 mm in diameter, with a very thin wall. We attempted to tape the right PA and hold its wall with forceps; however, the wall had begun to bleed. We attempted to clamp the PA and control the bleeding with sutures; however, as the artery wall was too fragile, we used a hemoclip as a partial clamp instead of taping to control the bleeding. After partially clamping the PA, we performed echocardiography from the operating field. Color Doppler indicated a blood velocity of 3.0 m/s at the right PA banding site, suggesting that the banding was effective. We performed the left PA banding using a hemoclip, as on the right side, and Doppler echo showed a blood velocity of 3.0 m/s in that location also (Fig. 2). After the operation, the patient’s hemodynamic status was initially stable, but echocardiography on postoperative day 1 showed dilatation distal to the right PA banding. Enhanced computed tomography on postoperative day 12 showed significant dilatation of the bilateral PA and descending aorta, which had not been observed before the operation (Fig. 3). At 21 days of age, the patient died of massive bleeding from the right thoracic cavity caused by rupture of the right PA. Subsequent genetic analysis indicated a de novo p.Thr 200 Pro (c.598A.C) mutation of the *TGFBR1* gene, confirming the diagnosis of LDS.

## Discussion

The clinical manifestation of LDS is variable, with features similar to those of Marfan syndrome. However, it is a more severe syndrome because life-threatening aortic dissection or rupture may occur in early childhood despite the aneurysm being smaller than that considered to be at high risk of rupture in Marfan syndrome [2]. Loeys et al. [3] reported that most vascular surgery for aortic aneurysms or dissections was performed at the mean age of 19.8 years in their study. Affected patients are at high risk of aortic dissection or rupture at an early age, but to our knowledge, surgery has never been performed on the great arteries in the neonatal period. Viassolo et al. [4] reported a fetal, not an operative, case of LDS with aortic root dilatation but no cardiac defect. In their case, the aortic aneurysm did not progress during pregnancy, and the ascending aorta dimension and appearance were normal at 32 weeks’ gestation. Cases of LDS complicated by simple congenital heart diseases, such as atrial septal defect (ASD) and PDA, have also been reported. Infants with PDA should undergo surgical ligation [5], whereas a large left-to-right shunt is unlikely to develop in those with ASD. Muramatsu et al. [6] reported a case of LDS with a large VSD, which was treated with PA banding on the 12th day. The main PA became subsequently more dilated, leading to a large aneurysm, so patch closure of the VSD and pulmonary arterioplasty were performed on the 42nd day. After the operation, progression of the main PA dilatation ceased. It is possible that after the PA banding, the turbulence of the blood flow in the distal site of the banding caused further progressive dilatation of the PA. Watanabe et al. [7] reported a case of LDS with VSD, bicuspid aortic valve, and dilatation of the ascending aorta and main PA. That infant underwent VSD patch closure on the 50th day and only mild dilatation of the ascending aorta was detected after the operation. Early radical surgery may be recommended; however, PA banding should represent a contraindication for neonatal LDS, as in our case. To our knowledge, the present case report represents the first documentation of LDS with complex congenital heart disease accompanied by an aortic arch anomaly. In this situation, the aortic arch would have to be repaired in the neonatal period if bilateral PA banding was not done initially. However, the arch repair might be extremely risky because of the fragility of the connective tissue; thus, we chose bilateral PA banding as the least invasive surgery to reduce the PA flow. Unfortunately, the bilateral PA and descending aorta dilated significantly as a result, and the patient died of a rupture of the massively dilated right PA.

In summary, the prognosis of this neonate with LDS and an interrupted aortic arch complex was extremely poor and the outcome of surgery reinforces the importance of considering the surgical indications carefully and possibly managing such patients with extremely complicated genetic anomalies conservatively.
